# The effects of modern housing on malaria transmission in different endemic zones: a systematic review and meta-analysis

**DOI:** 10.1186/s12936-024-05059-x

**Published:** 2024-08-07

**Authors:** Mukumbuta Nawa, Catherine Mupeyo-Mudala, Sylvia Banda-Tembo, Olatunji Adetokunboh

**Affiliations:** 1 Division of Epidemiology and Biostatistics, Stellenbosch University, Stellenbosch, Western Cape South Africa; 2 Department of Epidemiology and Biostatistics, Levy Mwanawasa Medical University, Lusaka, Zambia; 3 DSI-NRF Centre of Excellence in Epidemiological Modelling and Analysis (SACEMA), Stellenbosch University, Stellenbosch, Western Cape South Africa

**Keywords:** Modern housing, Traditional, Modern, Malaria-endemic zones

## Abstract

**Background:**

Modern housing has been shown to reduce the risk of malaria infections compared to traditional houses; however, it is unclear if the effects differ in different malaria transmission settings. This study evaluated the effects of modern housing on malaria among different endemic areas.

**Methods:**

Electronic databases, clinical trial registries and grey literature were searched for randomized controlled trials, cohort studies, case–control studies, and cross-sectional surveys on housing done between 1987 and 2022. Forest plots were done, and the quality of evidence was assessed using the Grading of Recommendations, Assessments, Development and Evaluation Framework.

**Results:**

Twenty-one studies were included; thirteen were cross-sectional, four were case–control and four were cohort studies. Cohort studies showed an adjusted risk ratio of 0.68 (95% CI 0.48–0.96), and cross-sectional studies indicated an adjusted odds ratio (aOR) of 0.79 (95%CI 0.75–0.83). By endemic transmission regions, the adjusted odds ratio in the high endemic settings was 0.80 (95%CI 0.76–085); in the moderate transmission regions, aOR = 0.76 (95%CI 0.67–0.85) and in the low transmission settings, aOR = 0.67 (95%CI 0.48–0.85).

**Conclusions:**

The evidence from observational studies suggests that there are no differences in the protective effects of modern houses compared to traditional houses on malaria by endemicity level. This implies that good quality modern housing protects against malaria regardless of the malaria transmission settings.

## Background

The fight against malaria has stalled in recent years partly due to the emergence of resistance to insecticides used in long-lasting insecticidal nets (LLINs) and indoor residual spraying (IRS), reduced investments and disruptions in interventions during the Coronavirus Disease of 2019 (COVID-19) pandemic [1]. Researchers have called for novel tools to enhance the fight against malaria for global elimination to be performed in line with the World Health Organization (WHO) Global Technical Strategy of eliminating 90% of incident cases and deaths by 2030 [1]. Two malaria vaccines have been approved so far, a malaria vaccine called RTS, S/AS01 (Mosquirix®) and another one called R21 (Matrix-M™), which have been added to the available tools to fight malaria [2, 3]. Others have called for community and human-centred approaches [4].

While the disease is raging on, researchers and policymakers are looking for solutions to emerging challenges. The case for housing infrastructure improvements in the fight against malaria, which was superseded by the discovery of chemical agents, has emerged [5]. Studies have shown that the risks and odds of malaria infection can be reduced by about 47% for those who dwell in modern houses compared to those who dwell in traditional houses [6]. Another study, a secondary analysis which analysed data from 15 Demographic and Health Surveys and 21 Malaria Indicators Surveys in sub-Saharan Africa found that modern houses were associated with reduced odds of malaria infection by about 9% (aOR 0.91 95%CI 0.85 -0.97) when compared to traditional houses [7]. Further, another study, a systematic review and meta-analysis which included 18 randomized controlled trials (RCTs) on the prevention of malaria and *Aedes*-transmitted diseases, found a reduced odds ratio of malaria in all settings of 0.63 (95%CI 0.39–1.01) [8]. Another study, a Cochrane review of RCTs, found that house modifications can reduce malaria prevalence at RR 0.68 (95%CI 0.57–0.82) [9]. The two systematic reviews focused on house modifications or improvements, such as the effects of fitments of screening or ceilings and closing of eaves, compared to controls that did not have those interventions [8].

While there is sympatry or co-existence of primary vectors, the primary *Anopheles* mosquito vectors that predominantly transmit malaria in highly endemic areas differ from those that predominantly transmit malaria in low endemic areas in terms of their feeding host preferences, resting behaviour, Entomological Inoculation Rates (EIR) and Sporozoite Infection Rates (SIR) [10, 11]. The effect of housing structures is likely to differ for high-endemic areas compared to low-endemic areas. This study, therefore, addressed this knowledge gap and can help government agencies target effective policies and interventions relevant to local settings.

## Methods

The study used the Preferred Reporting Items for Systematic Reviews and Meta-Analysis (PRISMA) guidelines to prepare a systematic review and meta-analysis [12]. The protocol was registered with the Prospective Register of Systematic Reviews (PROSPERO–ID 357186).

### Study settings

This review included studies from sub-Saharan Africa, South America, and Middle and East Asia and was stratified according to malaria-endemic zones.

### Inclusion and exclusion criteria

#### Types of studies

Studies included were RCT designs and observational studies such as cross-sectional surveys, case–control and cohort studies published between 1987 and June 2022 in line with the establishment of the Roll Back Malaria Initiative in 1987. All studies with clear effect measures (such as Odds Ratios, Incidence Rate Ratios, Prevalence Ratios and Indoor Vector Density Ratios, Entomological Inoculation Rate Ratios) were included, whilst those that were qualitative or without effect measures were excluded. Those without clear geographical areas where the studies were conducted were also excluded. Studies that meet the criteria for modern houses versus traditional houses but only compared components of house improvements such as iron roofs versus thatched roofs or brick walls versus mud walls were also excluded.

#### Type of participants

The study included studies that compared malaria occurrence in all types of residents, whether children under five years or adults or specific subsections of adults such as pregnant women.

### Interventions

Studies had to be clear that they compared modern housing structures against traditional or non-standard housing structures. Modern houses have finished wall and roofing materials; finished wall materials include cement, stone with lime or cement, bricks, cement blocks, covered adobe, and wood planks or shingles, while finished roofing materials include metal, wood, calamine or cement fibre, ceramic tiles, cement, and roofing shingles [7]. All other houses that do not have finished walls and roofing materials are considered traditional houses [7, 13]. This study did not include floor materials because they do not play a role in mosquito house entry. This definition of finished materials is not arbitrarily defined by the authors of this study but is in line with the demographic and health surveys as well as malaria indicator survey methodology guidelines [14].

### Type of outcome measures

Different studies measure malaria outcomes in different ways. Cross-sectional studies measure malaria prevalence diagnosed by blood slides using light microscopy regardless of symptoms. We did not include studies that measured malaria infection using rapid diagnostic tests (RDTs) due to their lack of specificity in detecting malaria. Cohort studies and RCTs measure malaria incidence. This study included prevalence and incidence as primary outcomes and analysed them separately by different endemic areas. Further, studies that compared entomological measures such as vector densities, human biting rates and entomological inoculation rates between modern houses and traditional houses were also included as secondary outcomes.

### Information sources

Major databases were searched for peer-reviewed journal articles on the subject, including Cochrane, MEDLINE (PubMed), Scopus, The Global Index Medicus and Web of Science. Peer-reviewed scientific conference proceedings, such as the American Society of Tropical Medicine and Hygiene, and The International Congress for Tropical Medicine and Malaria, were searched. Further, the study also searched clinical trial registries, including the WHO clinical trials registry and the American clinicaltrials.gov and grey literature.

### Search strategy

A literature search strategy was developed in Medline using Mesh subject headings combined with free text. The search strategy developed in Medline was adapted to other databases in collaboration with the University of Stellenbosch librarian and has been attached as supplementary material.

### Study records

The identified articles were imported into a citation reference manager called Endnote; Endnote was used to de-duplicate articles.

### Screening for eligibility

Rayyan QCRI Software was used to screen the articles for eligibility [15]. Three reviewers (NM, CMM and SBT) independently screened the titles and abstracts in Rayyan. Disagreements were resolved by discussion between the team members. (CMM and SBT), then read the full text for the selected articles and finalise the screening process with NM. OA supervised the screening process.

### Data extraction

Two reviewers (CMM and SBT) extracted data from selected studies into a pre-piloted data extraction form. The consensus was established between the two, and arbitration by the third reviewer (NM) when needed. The data points included authors, year of publication, sample size, study design, effect measures with 95% confidence intervals, type of participants, and geographical coverage.

#### Assessment of risk of bias in included studies

Three reviewers (NM, CMM and SBT) assessed the risk of bias in the studies in duplicate. The risk of bias for observational studies was assessed using the Risk of Bias for Non-Randomised Studies for Exposure (RoBINS-E) [16]. The risk of bias in the papers was reported as low risk, moderate risk, serious or critical risk based on the algorithm.

### Measures of treatment effects and associations

The outcome of this study was to establish the effects and measures of the association of modern houses on malaria cases (incidence and prevalence) stratified by low, moderate, and high endemic settings. The malaria endemicity settings of low, moderate and high were based on the WHO classification of the prevalence of *Plasmodium falciparum/Plasmodium vivax* of below 10% as low transmission, between 10 and 35% as moderate and above 35% prevalence as high transmission [17]. Clinical trials and cohort studies that report risk ratios were analysed and reported separately. At the same time, prevalence and case–control studies that report odds ratios were also analysed and reported separately.

### Unit of analysis issues

For follow-up studies such as RCTs and Cohort studies, Incidence Risk Ratios (IRR), Rate Ratios or Absolute Risk Differences were used to compare malaria incidence in modern houses versus traditional houses in different endemic settings. Where events occur below 10% in the samples, odds ratios were used as they are better estimates in rare events. In cross-sectional and case–control studies, the analysis unit used was odds ratios.

### Assessment of heterogeneity

In line with the Cochrane guidelines, heterogeneity in the studies was assessed using the *I*^*2*^ statistics in the meta-analysis, which is calculated by:

$${\text{I}}^{{2}} \, = \,\left( {\left( {{\text{Q}} - {\text{df}}} \right)/{\text{Q}}} \right) \, *{1}00$$

where Q is the Chi^2^ and df is the degree of freedom.

An *I*^*2*^ of 75–100% would be interpreted as considerable heterogeneity, 50–90% as substantial heterogeneity, 30–60% as moderate, and below 40% as unimportant [18].

### Assessment of reporting biases

Reporting bias was assessed using funnel plots where there were at least ten studies included in the meta-analysis.

### Data synthesis

A summary of how many articles were identified during the literature search, how many were excluded at what stage of the process, why they were excluded, and how many were finally included are presented in a flow diagram [19]. A descriptive table of included articles, where, when, authors, and effect sizes are presented. Forest plots were done of the analysis displaying pooled effect measures, 95% confidence intervals, p values, Chi-square, and *I*^*2*^ values. Meta-analyses were conducted among similar studies to find the pooled effect measures by endemic zone using RevMan for Windows (version 5.4) [20].

Similar study designs that reported the same measures of association and effect measures were used to create separate forest plots. Separate forest plots were run for each study design using the reported effect measure, whether risk ratio, rate ratio, absolute risk difference, or odds ratio low, moderate, and high endemic settings.

### Certainty of the evidence

The certainty of the evidence was assessed using the Grading of Recommendations, Assessment, Development and Evaluations (GRADE) framework [21]. Evidence was categorised as very low, low, moderate, and high quality. The certainty of evidence has been included in the GRADE table.

### Ethical considerations

The Department of Global Health research protocol panel at the University of Stellenbosch reviewed and approved this study. An exemption for review was obtained from the Health Research Ethical Committee at the University of Stellenbosch as it does not involve human subjects (HREC Reference number: X22/08/020).

## Results

**Fig. 1:**
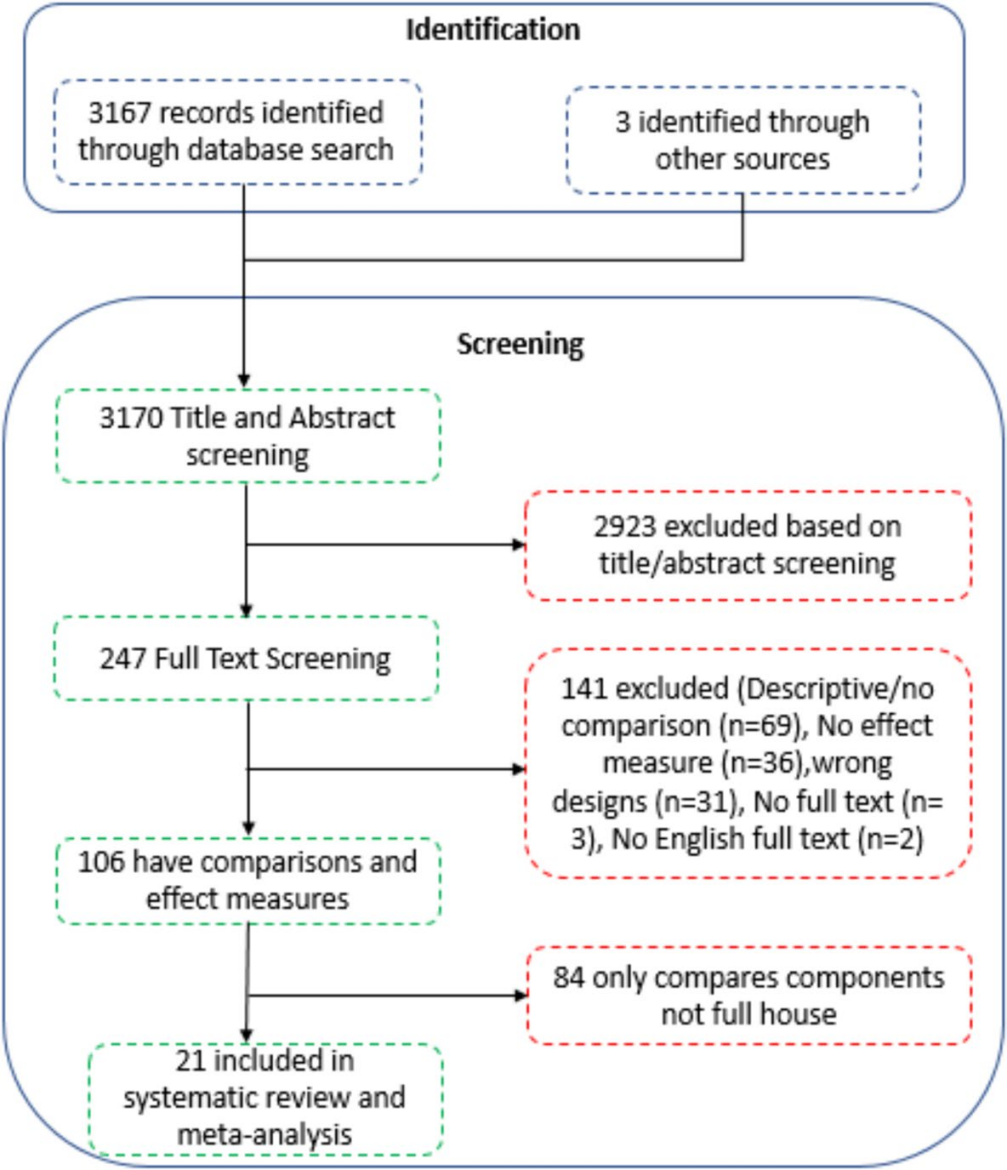
Inclusion flow chart

A total of 3,167 articles were collected from the database search, and an additional three were collected from grey literature, totalling 3,170. Following screening, 2,923 articles were excluded, and a full-text screening was done on 247 articles. A total of 141 were excluded on full-text screening, and 84 were excluded because they compared components and not comprehensive houses. Figure 1 shows the inclusion flow chart.

### Included studies

The majority of the studies included were cross-sectional study designs 13 (62%), case–control studies 4 (19%) and cohort studies 4 (19%). More than three-quarters of the studies were done in Africa 18 (86%), less than a fifth in Asia 3 (14%) and none in Latin America. Over half of the studies were cross-sectional surveys.

### Study settings

The majority of the studies done in Africa were done in high endemic settings 10/17 (59%), a third 4/17 (24%) from moderately endemic settings and 18% (3/17) in low endemic settings. Those from Asia were from moderate endemic settings in India and Pakistan.

### Characteristics of study participants

Among the studies included those that assessed malaria parasites among participants were 21 studies, and altogether, there were 234 262 participants. Table 1 gives a summary of the characteristics of the included studies.

**Table 1:** Characteristics of Included Studies

Author	Year	Continent	Country	Population	Endemicity	Design	Mal. Measure	Effect	Estimate	Lower CI	Upper CI	P value	Study Pop
Dahesh et al.	2009	Africa	Egypt	All age	Low	Case–control	Parasitaemia	Odds ratio	0.68	0.28	1.66		
Dlamini et al.	2017	Africa	Swaziland	All age	Moderate	Cross-Sec	Parasitaemia	Odds ratio	0.47	0.28	0.79		11,426
Ippolito et al.	2017	Africa	Zambia	All age	Moderate	Cross-Sec	Parasitaemia	Odds ratio	0.26	0.09	0.73	0.01	2788
Manyangadze	2022	Africa	Zimbabwe	All age	Low	Cross-Sec	Parasitaemia	Odds ratio	0.41	0.2	0.83		
Mohan et al.	2021	Asia	India	All age	Moderate	Cross-Sec	Parasitaemia	Odds ratio	0.91	0.77	1	0.01	70,671
Morakinyo et al.	2018	Africa	Nigeria	5–59 m	High	Cross-Sec	Parasitaemia	Odds ratio	0.71	0.56	0.93	0.01	6991
Mosha et al.	2020	Africa	Tanzania	0.5–14 y	High	Cross-Sec	Parasitaemia	Odds ratio	0.27	0.13	0.54	0.001	6918
Mundagowa et al.	2020	Africa	Zimbabwe	All age	Moderate	Case–control	Parasitaemia	Odds ratio	0.23	0.11	0.51	0.01	150
Nawa et al.	2020	Africa	Zambia	6–59 m	High	Case–control	Parasitaemia	Odds ratio	0.33	0.11	0.99	0.047	720
Nawa et al.	2019	Africa	Zambia	6–59 m	High	Cross-Sec	Parasitaemia	Odds ratio	0.4	0.34	0.71	0.001	19,320
Okiring et al.	2019	Africa	Uganda	Preg wom	High	Cohort	Incidence	IRR	0.76	0.69	0.87	0.001	753
Rek et al.	2018	Africa	Uganda	0.5–10 y	High	Cohort	HBR	IRR	0.27	0.17	0.42	0.001	384
Rek et al.	2018	Africa	Uganda	0.5–10 y	High	Cohort	HBR	IRR	0.52	0.36	0.73	0.002	384
Rugnao et al.	2019	Africa	Uganda	2–10 y	High	Cross-Sec	Parasitaemia	Prev Ratio	0.88	0.81	0.97	0.0008	8834
Salunkhe et al.	2019	Asia	India	All age	Moderate	Cross-Sec	Parasitaemia	Odds ratio	0.72	0.39	1.35		4954
Sikalima et al.	2021	Africa	Zambia	All age	High	Cross-Sec	Parasitaemia	Odds ratio	0.38	0.2	0.73	0.01	282
Smith et al.	2021	Africa	Namibia	All age	Moderate	Case–control	Parasitaemia	Odds ratio	0.5	0.36	0.69		1411
Snyman et al.	2015	Africa	Uganda	0–24 m	High	Cohort	Incidence	IRR	0.54	0.39	0.78	0.001	515
Tusting et al.	2016	Africa	Uganda	Mosq. Pop	Low	Cohort	HBR	IRR	0.53	0.4	0.69	0.001	124,746
Tusting et al.	2016	Africa	Uganda	All age	Low	Cohort	Incidence	IRR	0.93	0.68	1.28	0.67	2399
Tusting et al.	2016	Africa	Uganda	All age	Low	Cohort	Parasitaemia	Odds ratio	0.51	0.36	0.71	0.001	3367
Wolff et al.	2001	Africa	Malawi	0–5 y	High	Cross-Sec	Parasitaemia	Odds ratio	0.73	0.36	1.41		318
Zaidi et al.	2015	Asia	Pakistan	All age	Moderate	Cross-Sec	Parasitaemia	Odds ratio	0.65	0.39	1.1		1623
Tusting et al.	2017	Africa	SSA	0–5 y	All	Meta-analysis	Parasitemia	Odds ratio	0.91	0.85	0.97	0.003	139 318

*y* years; *m* months; *mosq. *Pop mosquito population; *preg. Wom* pregnant women; *cross-sect* cross-sectional study; *vector den* vector density; *IRR* Incidence rate ratio; *SSA* sub-Saharan Africa

### Housing characteristics

All 21 studies included in this review compared modern houses against traditional houses. Traditional houses were different in Africa and Asia but followed the DHS classification while modern houses were made of brick walls and iron/tiled ceramic roofs.

### Primary outcomes

The primary outcomes reported in the included studies were malaria prevalence and incidence depending on the study design. A total of 17 outcomes of the interventions reported the prevalence of malaria parasites in the respondents. A total of four outcomes were reported on malaria incidence. Some studies reported more than one outcome.

### Secondary outcomes

Two entomological studies reported indoor resting vector densities, while two other studies reported human biting rates. No entomological study among the included studies linked entomological inoculation rate and housing structures.

### Excluded studies

A total of 84 studies were excluded on the basis that though they had effect measures on housing structures comparing malaria in traditional versus modern structures, they only compared components of houses such as thatched roof versus iron/tiled roof, ceiling versus no ceiling, closed eaves versus open eaves or mud walls versus brick walls.

#### Risk of bias assessment

Twenty of the included studies were observational and assessed for risk of bias using the Risk of Bias in Non-Randomised Studies of Exposure (RoBINS-E) Tool [16]. Five of the studies had a serious risk of bias arising from recall bias due to prolonged periods assessed [22, 23], risk of selection bias [24] and confounding due to the use of unadjusted odds ratios in the studies [25, 26]. Ten included studies had moderate concerns, mainly arising from residual confounding in cross-sectional and case–control studies, even after multivariate regression adjustment. Four had a low risk of bias mainly because they were cohort studies [13, 27–29]. A summary of the risk of bias assessment for the observational studies is shown in Table 2.

**Table 2:** Risk of bias in included studies

Risk of bias in observational studies											
Author	Year	Design	D1: Confounding	D2: Exposure measurement	D3: Participant Selection	D4: Post exposure interventions	D5: Missing data	D6: Measurement of outcome	D7: Reporting bias	Overall rating	Comments
Dahesh et al.	2009	CC	Y	PY	N	N	No Info	N	N	Serious risk	Used unadjusted odds ratio
Dlamini et al.	2017	XS	PY	PY	N	N	No Info	N	N	Moderate risk	
Ippolito et al.	2017	XS	PY	PY	N	N	No Info	N	N	Moderate risk	
Manyangaze et al.	2022	XS	PY	PY	N	N	No Info	PY	N	Serious risk	Malaria cases were assessed over two decades. Recall bias
Mohan et al.	2021	XS	PY	PY	N	N	No Info	Y	N	Serious risk	High chance of recall bias of malaria in past two year
Morakinyo et al.	2018	XS	PY	PY	N	N	No Info	N	N	Moderate risk	
Mosha et al.	2020	XS	PY	PY	N	N	No info	N	N	Moderate risk	
Mundagowa et al.	2020	CC	PY	PY	N	N	N	N	N	Moderate risk	
Nawa et al.	2020	CC	PY	PY	N	N	N	N	N	Moderate risk	
Nawa et al.	2019	XS	PY	PY	N	N	N	N	N	Moderate risk	
Okiring et al.	2019	CH	N	N	N	N	N	N	N	Low risk	
Rek et al.	2018	CH	N	N	N	N	N	N	N	Low risk	
Rugnao et al.	2019	XS	PY	PY	N	N	No Info	N	N	Moderate risk	
Salunkhe et al.	2019	XS	PY	PY	N	N	No Info	N	N	Moderate risk	
Smith et al.	2021	CC	PY	PY	N	N	No Info	PY	N	Moderate risk	
Snyman et al.	2015	CH	N	N	N	N	No Info	N	N	Low risk	
Tusting et al.	2016	CH	N	N	N	N	No Info	N	N	Low risk	
Wolff et al.	2001	XS	PY	PY	PY	N	No Info	N	N	Serious risk	A committee selected improved house beneficiaries
Zaidi et al.	2015	XS	Y	PY	N	N	No info	N	N	Serious risk	Used unadjusted odds ratio
Tusting et al.	2017	Meta-analysis	PY	PY	N	N	No info	N	N	Moderate risk	

*CC* Case–Control, *XS* Cross-Sectional, *CH* Cohort, *Y* Yes, *PY* Probably Yes, *N* No, *No Info* No Information

### Effects and associations of interventions/ exposures on outcomes

The overall association of modern houses on the risk of malaria parasitaemia compared to traditional housing among cross-sectional surveys using the adjusted odds ratios reported in the individual studies was a reduction in the adjusted odds ratio of 0.79 (95%CI 0.75–0.83). The overall heterogeneity was high at I^2^ = 66.2% and was statistically significant (P value < 0.001), implying that there were significant differences in the association of modern housing in different individual studies. Table 3 summarises the pooled measures of associations.

**Table 3:** Summary of pooled measures of association

No	Study design	No. of studies	Endemicity	Outcome	Association type	Measure (95% CI)	Heterogeneity overall	Heterogeneity subgroups
1	Cross-Sectional	41	All	Parasitaemia	aOR	0.79 (0.75–0.83)	0.66	
		24	High	Parasitaemia	aOR	0.80 (0.76–0.85))		0.72
		7	Moderate	Parasitaemia	aOR	0.76 (0.67–0.85)		0.74
		10	Low	Parasitaemia	aOR	0.67 (0.48–0.85)		0
2	Cross-Sectional	33	All	Parasitaemia	uOR	0.28 (0.27–0.29)	0.94	
		18	High	Parasitaemia	uOR	0.34 (0.33–0.36)		0.95
		5	Moderate	Parasitaemia	uOR	0.14 (0.12–0.17))		0.78
		10	Low	Parasitaemia	uOR	0.20 (0.16–0.24)		0.75
3	Case–Control	2	All	Parasitaemia	aOR	0.52 (0.38–0.70)	0	0
		1	High	Parasitaemia	aOR	0.33 (0.11–0.99)		–
		0	Moderate	Parasitaemia	aOR	–		–
		1	Low	Parasitaemia	aOR	0.54 (0.39–0.74)		–
4	Case–Control	2	All	Parasitaemia	uOR	0.33 (0.06–1.75)	0.71	–
		0	High	Parasitaemia	uOR	–		–
		0	Moderate	Parasitaemia	uOR	–		–
		2	Low	Parasitaemia	uOR	0.33 (0.06–1.75)		0.71
5	Cohort	2	All	Incidence	aRR	0.68 (0.48–0.96)	0.71	–
		2	High	Incidence	aRR	0.68 (0.48–0.96)		0.71
		0	Moderate	Incidence	aRR	–		–
		0	Low	Incidence	aRR	–		–
6	Cohort	2	All	Incidence	uRR	0.89 (0.70–1.14)	0	–
		2	High	Incidence	uRR	0.89 (0.70–1.14)		0
		0	Moderate	Incidence	uRR	–		–
		0	Low	Incidence	uRR	–		–
7	Cohort	2	All	Parasitaemia	uOR	0.63 (0.41–0.97)	0.69	–
		2	High	Parasitaemia	uOR	0.63 (0.41–0.97)		0.69
		0	Moderate	Parasitaemia	uOR	–		–
		0	Low	Parasitaemia	uOR	–		–
8	Cohort	2	All	Human Biting Rate	uRR	0.53 (0.43–0.65)	0	–
		2	High	Human Biting Rate	uRR	0.53 (0.43–0.65)		0
		0	Moderate	Human Biting Rate	uRR	–		–
		0	Low	Human Biting Rate	uRR	–		–

When the effect of modern housing was stratified by endemicity, the effect in the high endemic zones was at an odds ratio of 0.80 (95%CI 0.76–0.85) and was statistically significant. The heterogeneity in the high endemic zone was high at I^2^ = 0.72.5% and statistically significant. The association of modern housing in the moderate malaria endemic zones compared to traditional housing was found to be statistically significant at odds ratio 0.76 (95%CI 0.67–0.85) with high heterogeneity at 74.4%. There was only one study done in India [22] while the rest were from Africa. In the low endemic zone, the association of modern housing compared to traditional housing also showed a significant reduction in malaria infections with an odds ratio of 0.67 (95%CI 0.45–0.85). There was an overlap in the confidence intervals of the odds ratios across the high, moderate and low endemic transmission areas indicating no statistical differences in the effects of modern houses on malaria compared to traditional houses.

Further, the study conducted a meta-analysis of cross-sectional studies using unadjusted odds ratios from reported actual numbers of infections and total participants included in studies. The pooled measure of association was an odds ratio of 0.28 (95%CI 0.27–0.29, I^2^ = 94.5%) This association was more than the one calculated from adjusted odds ratios, probably because of confounding from other factors that were not adjusted for in the analysis using unadjusted odds ratios. Similarly, the effects of modern housing compared to traditional housing in the low transmission settings was more uOR 0.20 (95%CI 0.16 – 0.24) compared to the moderate and high transmission settings (uOR of 0.14, 95%CI 0.12 – 0.17 and 0.34, 95%CI 0.33–0.36, respectively).

The study further assessed the associations of modern housing compared to traditional housing using case–control studies that reported adjusted odds ratios by endemic zones. There were only two studies in the meta-analysis, one done in Zambia and the other in northern Namibia. The overall effect of modern housing compared to traditional housing was an odds ratio of 0.52 (95%CI 0.38–0.70, I^2^ = 0%, P value = 0.40), which shows that modern housing had a statistically significant effect in reducing the risk of malaria compared to traditional housing. In terms of endemicity, only one case–control study was included in the high endemic region, and the effect measure was an adjusted odds ratio of 0.33 (95%CI 0.11–0.99). No studies were included that were done in the moderately endemic regions. In contrast, one study was included in the low malaria endemic region, and the effect measure was an odds ratio of 0.54 (95%CI 0.39–0.74). Due to the few studies included in the meta-analysis, the heterogeneity was low.

This review further analysed case–control studies that reported an unadjusted number of malaria infection events against totals and conducted a meta-analysis. Only two studies were included, one from Egypt and another from Zimbabwe, which were both in low transmission settings. The pooled measure of association was an odds ratio of 0.33 (95%CI 0.06–1.75, I^2^ = 71% and P value = 0.19). This association was not statistically significant because of a wide confidence interval, few studies, and likely confounding from the unadjusted odds ratios used.

Further analysis of observational studies was done using cohort studies that compared adjusted incidence (risk) ratios among residents of modern houses against traditional houses. Two cohort studies compared the adjusted risk ratios in modern houses to traditional houses, both done in Uganda (13, 28). There was a reduced Incidence Risk Ratio (IRR) of 0.68 (95%CI 0.48–0.96, I = 71%, P value = 0.06). The risk reduction was statistically significant based on the confidence intervals. Still, the heterogeneity was not significant, probably because of the few studies and that they were done in the same country.

A meta-analysis of cohort studies that reported unadjusted Incidence Risk Ratios found only two studies from Uganda and pooled risk ratios of 0.89 (95%CI 0.70–1.14) [13, 28]. This effect was not statistically significant, unlike the ones done from the same country, Uganda, in similar settings that reported adjusted Risk Ratios.

The same two cohort studies done in Uganda that reported non-significant risk ratios also reported unadjusted odds ratios, which were statistically significant (Odds Ratio 0.63 (95%CI 0.41–0.97) when pooled in a meta-analysis [27, 30].

In cohort studies, the study further explored the association of modern housing compared to traditional housing using mosquito vectors’ Human Biting Rate (HBR). The same two studies from Uganda reported the unadjusted risk ratio using HBR (RR 0.53 (95%CI 0.43–0.65) [27, 30].

### Quality of evidence (GRADE)

The studies included in this review provide low-quality evidence from cohort studies and low to very low evidence from cross-sectional and case–control studies [31]. Table 4 shows the summary for the certainty of evidence using the GRADE Approach.

**Table 4:** Quality of Evidence from Observational Studies

Certainty assessment								No. of patients		Effect		Certainty
Outcomes	No of Studies	Study design	Risk of bias	Inconsistency	Indirectness	Imprecision	Other considerations	Modern housing	Traditional housing	Relative (95% CI)	Absolute (95% CI)	
Parasitaemia (Adjusted ORs)	41	Observational studies (cross-sectional)	Serious^a^	Not serious^b^	Not serious^c^	Not serious^d^	None	12,143/ 111,103 (10.9%)	30,935/ 123 159 (25.1%)	**OR** 0.365 (0.358 –0.374	**64 fewer per 1,00** (from 64 to 62 fewer)	⨁⨁◯◯ Low
Parasitaemia (Unadjusted ORs)	33	Observational studies (cross-sectional)	Very serious^a^	Serious^e^	Not serious^c^	Serious^f^	None	5550/ 41,272 (13.4%)	26,451/ 97,926 (27.0)	**OR** 0.42 (0.40–0.43	**58 fewer per 1,00** (from 60 to 57 fewer)	⨁◯◯◯ Very low
Parasitaemia (Adjusted ORs)	2	Observational studies (case–control)	Serious	Not serious^b^	Not serious^c^	Not serious^d^	None	979 cases,	1282 controls	**OR 0.52 **(0.38 to 0.70)	–	⨁⨁◯◯ Low
								–	31.0%		**121 fewer per 1000** (from 164 to 71 fewer)	
Parasitaemia (Unadjusted ORs)	2	Observational studies (case–control)	Very serious^a^	Serious^e^	Not serious^c^	Serious^g^	None	51 cases,	80 controls	**OR 0.33 **(0.06 to 1.75)	**–**	⨁◯◯◯ Very low
								–	42.0%		**227 fewer per 1000** (from 378 to 139 fewer)	
Incidence Rate Ratios (Adjusted)	2	Observational studies (cohort)	Serious^h^	Not serious^b^	Not serious^c^	Not serious	None	73/174 (42.0%)	463/957 (48.4%)	**RR 0.68** (0.48 to 0.96)	**155 fewer per 1000** (from 252 to 19 fewer)	⨁⨁◯◯ Low

CI: confidence interval; OR: odds ratio; RR: risk ratio

^a^Downgraded for serious risk of bias: All studies were observational with a significant risk of bias

^b^Most studies observed a protective effect of modern housing compared to traditional housing

^c^No serious indirectness: These studies were conducted in different settings and countries. The findings are generalizable in other places

^d^No serious imprecision: The overall effect was statistically significant and clinically important

^e^Few studies observed a protective effect of modern housing compared to traditional housing

^f^The overall effect was not statistically significant

^g^The overall effect was not statistically significant and with wide confidence intervals

^h^Downgraded for serious risk of bias: All studies were observational and non-randomized

## Discussion

This systematic review and meta-analysis sought to find the effects and measures of association between modern housing and malaria infections in different malaria endemic zones. Previous meta-analyses, particularly non-Cochrane studies that included sufficient observational studies, found high heterogeneity in the measures of associations between housing structures and malaria parasitaemia [32]. The high heterogeneity may arise from differences from not only study designs but also in endemic settings. This study’s findings indicate that modern housing provides reduced risks of malaria infection as measured in different study designs including cohort (IRR 0.68, 95%CI 0.48–0.96), case–control (aOR 0.52, 95%CI 38–0.70) and cross-sectional studies (aOR 0.79, 95%CI 0.75–0.83). These findings are in agreement with other systematic reviews and meta-analyses on modern housing compared to traditional housing which showed that modern housing reduced the risk of malaria infections [7, 32, 33]. This study did not find RCTs that met the inclusion criteria, therefore it did not include any RCTs; existing RCTs and meta-analyses of RCTs consisted of studies that compared house improvements such as iron roofs versus traditional roofs, brick walls versus traditional walls and other interventions such as window and door screens, and eave closure versus no intervention [8, 9].

This study, therefore, only included observational studies, such as cohort, case–control and cross-sectional studies; the results show that modern houses that include both iron roofs and brick walls reduce malaria risk and indoor vector densities with very few showing that the measures of association are not statistically significant [25, 27, 30, 34]. However, socio-economic factors such as wealth, education, nutritional status and health status are also associated with living in modern houses compared to living in traditional houses [35]. Therefore, even when some socio-economic factors were adjusted for in the observational studies included in the review and meta-analysis, residual confounding was still an important factor. As such, the results were considered to have low to very low certainty of evidence using the GRADE system. They must be interpreted with caution [21].

From the meta-analysis, four cohort studies were all done in Uganda and were categorized as high-endemic settings [13, 27–29]. Of the four case–control studies included, three were in low transmission settings [25, 36, 37], and only one was in high endemic settings [6]. So, the comparisons could only be made using cross-sectional studies where there were studies in all endemic settings, which allowed us to do comparisons using the same measures of association. The risk reduction of malaria in modern housing was not statistically different in high, moderate and low malaria transmission settings when comparing confidence intervals of the pooled odds ratios using adjusted odds ratios. Similarly, cross-sectional studies that reported unadjusted odds ratios also showed that the association between modern housing and malaria infection compared to traditional housing was not statistically different across high, moderate and low transmission settings, however, the effect measure was significantly lower when assessed using unadjusted odds ratios compared to adjusted odds ratios. This study based its conclusion on adjusted odds ratios because unadjusted odds ratios have statistical noise and confounding which were not adjusted for. It is possible that factors such as wealth status and residing in rural areas among others could have contributed to the overestimation of the effects of modern housing in unadjusted odds ratios i.e., more poor people in Africa tend to live in poor housing structures in rural areas, so not adjusting for these factors (wealth and residence location) may overestimate the effects of modern housing. One study from India showed a very minimal risk reduction, which was not statistically significant, probably because it only measured malaria in the adult population aged 45 years and above, which is different from children aged below five years and the general population, which most cross-sectional studies in Africa measure malaria in [22]. The authors did not find any systematic reviews or meta-analyses that compared malaria risk of infection in different endemic areas to compare with this study.

Modern housing has a biological plausibility of being more effective compared to traditional housing; from an entomological perspective, high transmission settings have higher entomological inoculation rates (EIRs) [38], so people get bitten many times by infected mosquitoes, and you would expect residents of traditional houses that do not impede mosquito entry to have a higher probability of infections compared to people in modern houses [39]. Conversely, as the EIRs reduce in moderate and low endemic transmission settings, you would expect a dose–response-like effect of reduced measures of association in moderate and low endemic settings [38]. So, based on the dose–response-like associations using higher EIRs in higher endemic settings and lower EIRs in moderate and low malaria endemic settings, it would be expected that measures of association of modern housing and risk of malaria would be higher in high endemic settings compared to low endemic settings. However, the effect was similar in all endemic transmission areas.

From an immunological perspective, those who get bitten more times in the higher transmission settings develop acquired immunity and can fend off infections and clinical disease even when bitten by infected mosquitoes multiple times [40]. Conversely, those in low transmission settings may not have had frequent bites enough to confer acquired immunity; for example, the EIR in some places in Uganda may be as high as 310 infective bites per person per year, whilst, in low transmission settings, such as Botswana, Namibia and the Southern parts of Zambia, the EIRs are below 1.6 infective bites per person per year [38]. A person bitten by an infective mosquito less than twice a year is less likely to develop acquired immunity than another who gets bitten by infective mosquitoes 310 times a year.

Elsewhere, policymakers and managers of malaria programmes have noted the reduced effects and associations of other interventions, such as LLINs and IRS [41]. Despite the low to very low quality of evidence available, the findings of this study may, therefore, be of interest in providing evidence for improved housing in fighting malaria in different endemic settings. Improving housing to modern standards to prevent malaria can be an addition to the tools available in the fight against malaria, especially now as the fight against malaria garner towards its elimination by 2030.

## Limitations

The main limitation of this study was that there were no high-quality evidence studies such as RCTs. Moderate-quality evidence from cohort studies was also not available in all endemic settings, so it mainly relied on low to very low-quality evidence from cross-sectional studies which are prone to bias and confounding. Further, malaria was measured in different populations, in under-five children in some studies such as Malaria Indicator Surveys, in the general populations in some surveys in low transmission areas such as Egypt and Zimbabwe and in people over 45 years in India. In addition, modern housing characteristics were not standardized as it included modern brick walls and iron or roof tiles; some variations in the designs can affect their effects on malaria such as ensuring that eaves are closed or doors are tightly fitted in a standardized way as would happen in randomized controlled trials.

## Conclusion

The currently available evidence on measures of association and effects of modern houses compared to traditional houses on malaria transmission in different endemic transmission settings is limited to low and very low-quality evidence. The evidence suggests that the risk reduction associated with modern housing compared to traditional housing structures is not significantly different in low, moderate and high transmission settings. Further, evidence from cohort studies done in high-transmission settings shows that modern houses may have the benefit of reducing the risk of malaria transmission and indoor vector densities.

### Implications for research

More research is needed to generate high-quality evidence in low and moderate endemic settings regarding the effects of house improvements in different endemic settings.

### Implications for practice

In all malaria-endemic areas, house improvements may be one of the additional tools for policymakers and programme managers to consider implementing in malaria programmes.

## Data Availability

No datasets were generated or analysed during the current study.
